# Sleep quality relates to emotional reactivity via intracortical myelination

**DOI:** 10.1093/sleep/zsaa146

**Published:** 2020-08-08

**Authors:** Nicola Toschi, Luca Passamonti, Michele Bellesi

**Affiliations:** 1 Department of Biomedicine and Prevention, University of Rome Tor Vergata, Rome, Italy; 2 Athinoula A. Martinos Center for Biomedical Imaging, Department of Radiology, Massachusetts General Hospital and Harvard Medical School, Boston, MA; 3 Consiglio Nazionale delle Ricerche (CNR), Istituto di Bioimmagini e Fisiologia Molecolare (IBFM), Milan, Italy; 4 Department of Clinical Neurosciences, University of Cambridge, Cambridge, UK; 5 IRCCS San Camillo Hospital, Venice, Italy; 6 School of Physiology, Pharmacology and Neuroscience, University of Bristol, Bristol, UK

**Keywords:** brain, HCP, myelin, MRI, sleep

## Abstract

A good quality and amount of sleep are fundamental to preserve cognition and affect. New evidence also indicates that poor sleep is detrimental to brain myelination. In this study, we test the hypothesis that sleep quality and/or quantity relate to variability in cognitive and emotional function via the mediating effect of interindividual differences in proxy neuroimaging measures of white matter integrity and intracortical myelination. By employing a demographically and neuropsychologically well-characterized sample of healthy people drawn from the Human Connectome Project (*n* = 974), we found that quality and amount of sleep were only marginally linked to cognitive performance. In contrast, poor quality and short sleep increased negative affect (i.e. anger, fear, and perceived stress) and reduced life satisfaction and positive emotionality. At the brain level, poorer sleep quality and shorter sleep duration related to lower intracortical myelin in the mid-posterior cingulate cortex (*p* = 0.038), middle temporal cortex (*p* = 0.024), and anterior orbitofrontal cortex (OFC, *p* = 0.034) but did not significantly affect different measures of white matter integrity. Finally, lower intracortical myelin in the OFC mediated the association between poor sleep quality and negative emotionality (*p* < 0.05). We conclude that intracortical myelination is an important mediator of the negative consequences of poor sleep on affective behavior.

**Statement of Significance**

To properly function, the brain needs myelin, an electrically insulating structure that surrounds axonal fibers. Previous animal work demonstrated that sleep disruption is detrimental for myelin, suggesting that one of the functions of sleep is to support myelin formation and maintenance. Sleep disruption is also commonly associated with cognitive and emotional deficits although the possible pathophysiological role of myelin in this association has not been ascertained yet. Here we demonstrate that poor sleep quality or short sleep duration are associated with lower myelin content in the human neocortex. We also show that intracortical myelin in the anterior orbitofrontal cortex mediates the association between poor sleep and negative emotionality, which can mechanistically explain how poor sleep influences affective behavior.

## Introduction

Sleep is a naturally recurring state of the brain and body that occupies about one third of our lives. Although its physiological functions remain to be fully elucidated, it is undebatable that chronic short sleep or poor quality of sleep lead to cognitive and emotional problems, ranging from impaired vigilance to increased risk for neuropsychiatric conditions [1]. The negative consequences of even a few hours of sleep deprivation can span across several cognitive domains including attention, memory, and perception [2]. Sleep loss also deteriorates emotional control, with increased intolerance to stress, higher impulsivity, augmented negative emotionality, and reduced positive affect [3–5].

The brain mechanisms underlying these effects are scarcely understood, although findings from animal and human research have suggested that modifications in myelin sheaths may be crucial mediators of poor sleep [6–10]. Acute or chronic sleep loss in rodent models also down-regulates several brain transcripts related to myelin function [6, 11, 12]. More specifically, our recent work has shown that ~5 days of sleep restriction in mice reduce myelin thickness and increase the length of Ranvier’s nodes in highly myelinated white matter tracts [7, 10]. Such changes have a profound impact on reducing the velocity of the signal propagation along axons [13], which in turn may represent the neurophysiological basis of the cognitive and emotional problems resulting from sleep deprivation.

In humans, the microstructural integrity of white matter can be indirectly assessed via fractional anisotropy (FA), a magnetic resonance imaging (MRI) index derived from diffusion tensor imaging (DTI). FA estimation is based on the assumption that axonal and myelin membranes limit the diffusion of water molecules within differentially oriented fiber tracts [14]. While FA (as well as other DTI-derived indices) is a sensitive marker of white matter integrity, it is unspecific for myelination as other factors like axon diameter, fiber density, and cellular organization concur to determining its value [14]. Recently, new and more sophisticated models have been developed to partially disentangle the different sources of variability in the diffusion signal including the neurite orientation and dispersion density imaging (NODDI) [15–17].

Thus far, several studies have investigated how interindividual FA variability relates to sleep quantity and quality in humans. One study in young healthy participants (*n* = 21, age range: 22.1 ± 2.1 years) reported widespread FA decreases in several brain regions after 23 h of wakefulness and a significant association between sleepiness levels after sleep deprivation and FA reductions [8]. A second study (*n* = 32, age range: 19–25 years) found that individuals who were cognitively more susceptible to sleep deprivation had lower FA in multiple brain regions relative to less vulnerable people [18]. Similarly, the FA in the white matter underneath different frontoparietal regions positively related to subject-specific resilience to sleep deprivation, as quantified via a psycho-motor vigilance test [19]. More recently, a DTI study in *n* = 448 community-dwelling adults (age-range: 69.2 ± 5.1 years) showed that poor sleep quality was linked to globally lower FA and overall higher axial and radial diffusivity (RD) in frontal white matter tracts [9]. Consistent with these findings, Kocevska and collaborators observed that sleep quality measured via actigraphy positively related to white matter microstructural integrity, particularly in the cingulum and anterior forceps of the corpus callosum in middle-age and older people (*n* = 1,001, 59.3 ± 7.9 years) [20]. However, these results were not confirmed in another study using a larger cohort of participants (*n* = 2,529, age-range: 56 ± 6 years) [21]. While most of the earlier diffusion imaging studies explored the relationship between FA and sleep measures, a notable recent work used quantitative anisotropy (QA), a new measure that captures more nuanced differences in the microstructure of the white matter (e.g. axonal density). Via this innovative approach, the more compact axonal pathways within the default-mode network, the control-execution network and the salience network were associated with greater resilience to mood alterations after one night of sleep deprivation [22].

Relative to the abundance of studies assessing the links between white matter integrity and sleep quality/quantity, research investigating the role of intra-cortical myelin on sleep remains limited, especially in humans. This has probably depended on the challenges inherent to quantifying the intracortical myelin content in vivo. However, recent MRI studies have shown that the T1/T2 signal ratio could be used as a reliable, although relative and indirect, in vivo estimator of intracortical myelin [23]. In particular, the in vivo T1/T2 signal ratio compares well to ex vivo quantifications of intracortical myelin [24, 25], for example, in brain tissues from patients with multiple sclerosis (a common demyelinating disorder) [23, 26, 27].

The T1/T2 ratio has also been successfully employed to track the intracortical myelin changes that occur across the lifespan [26–28]. Similarly, the T1/T2 ratio recapitulates the well-known pattern of cortical myelination, which occurs first in primary sensory-motor areas (e.g. visual cortex), next in associative regions such as the temporal and parietal lobes, and ultimately in the prefrontal cortex [28]. Subject-specific variability in the T1/T2 ratio has also been linked to individual differences in cognitive performances and personality traits [26, 29]. This suggests that the T1/T2 ratio is a valid proxy to study the impact of quality and quantity of sleep on the human cortical myelo-architecture [26].

In this study, we exploited a large and neuropsychologically well-characterized sample of healthy individuals (*n* = 974) to test the hypothesis that self-reported quality and quantity of sleep related to: (1) cognitive performance and emotional behavior and (2) white matter integrity and intracortical myelin content. Furthermore, we tested whether the effects of sleep quantity and quality on cognitive performance and emotional behavior were mediated by interindividual differences in brain myelination.

Our prediction was that chronic short sleep and/or poor sleep quality were linked to: (1) scarce cognitive performance and higher negative emotionality (and/or lower positive affect) and (2) lower intracortical myelin and lower white matter microstructural integrity, globally or in specific frontotemporal cortices [9, 19]. We also tested the hypothesis that chronic sleep alterations were linked to poorer cognitive performance and/or altered emotional reactivity via the mediating role of reduced levels of myelin. To these ends, we used: (1) self-report questionnaires of sleep quality/quantity and emotional behavior (e.g. anger, anxiety, stress, and positive affect); (2) neuropsychological measures of attention, episodic memory, reward-based impulsivity, visuospatial skills, and information processing speed; (3) DTI- and NODDI-based indices of white-matter integrity; and (4) T1/T2-based estimates of intracortical myelin.

## Methods

The participants included in this study were drawn from the Human Connectome Project (HCP), a large international consortium that has provided access to high-quality behavioral and neuroimaging measures [30] (https://www.humanconnectome.org/). Out of the approximately *n* = 1,200 original sample from the HCP dataset, we employed all participants for which a T1/T2-derived myelin map as well as valid diffusion MRI data were available, resulting in *n* = 974 participants. See Table 1 for the demographics of the sample included in this study.

**Table 1.** Demographics of the sample (*n* = 974 participants) included in the study

	Mean ± standard deviation
Age (years)	28.7 ± 3.7
Sex (M/F)	444/530
Education (years)	14.9 ± 1.8
Body mass index	26.4 ± 5.2
Systolic blood pressure (mmHg)	123.7 ± 13.9
Diastolic blood Pressure (mmHg)	76.7 ± 10.6
Race (%)	Asian/Natural Hawaiian/Other Pacific Islands: 5.6% Black or Afrin American: 15.1% White: 75.1% More than one: 2.5% Unknown or not reported: 1.5%
Ethnicity (%)	Hispanic/Latino: 8.8% Not Hispanic/Latino: 90.1% Unknown or not reported: 1%
Handedness (%)	Right-handed: 88.1% Left-handed: 7% Mixed: 5%

### Sleep measures

**Figure 1.**
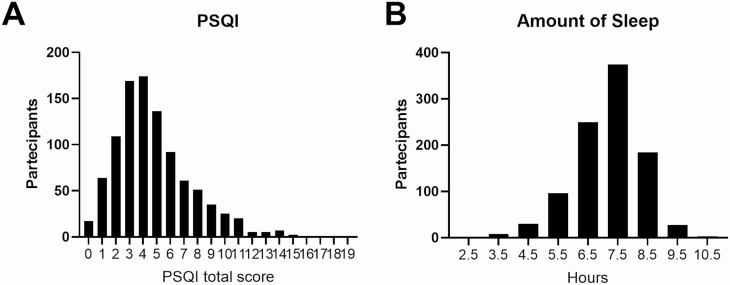
Frequency distribution of PSQI total score (A) and sleep amount (B).

Before MRI scanning, participants were asked to complete the Pittsburgh Sleep Quality Index (PSQI) questionnaire, a self-reported measure of sleep quality and amount of sleep in hours [31]. The PSQI is calculated by adding seven subscores to a total score ranging from 0 to 21, where lower scores denote better sleep quality. The total PSQI score and one of its subitems, namely the amount of sleep (i.e. the average sleep duration reported by the subject over the last month), were included in the statistical models that examined the associations with the behavioral and MRI measures. A summary of the PSQI scores (total and for each component) for the 974 HCP participants is provided in Table 2 and Figure 1.

**Table 2.** PSQI data in the sample (*n* = 974 participants) included in the study

	Mean ± standard deviation
PSQI total score	4.8 ± 2.8
PSQI component 1 Subjective sleep quality	0.9 ± 0.6
PSQI component 2 Sleep latency	1 ± 0.8
PSQI component 3 Sleep duration	0.6 ± 0.8
PSQI component 4 Sleep efficiency	0.4 ± 0.8
PSQI component 5 Sleep disturbance	1.1 ± 0.5
PSQI component 6 Use of sleep medication	0.2 ± 0.7
PSQI component 7 Daytime dysfunction	0.6 ± 0.6
PSQI Amount of sleep (h)	6.8 ± 1.1

### Cognition and emotional behavior analysis

Cognition and emotional behavior were assessed with a battery of cognitive and emotional tests and questionnaires, at the same timepoint of the MRI scans. The cognitive tests evaluated visual and verbal episodic memory, inhibitory control, sustained attention, cognitive flexibility, fluid intelligence, processing speed, reading abilities, vocabulary, spatial orientation, working memory, and delay discounting (i.e. reward impulsivity). Questionnaires measures assessed negative affect (sadness, fear, and anger), stress and psychological well-being (positive affect, life satisfaction, meaning, and purpose), social relationships, and self-efficiency. Age-adjusted cognitive and emotional scores were used to evaluate the relationships between cognition/emotion and PSQI using bivariate correlations with Bonferroni’s correction for multiple comparisons. See Supplementary File for a list of all the tests used in this study.

### MRI scanning protocols

All MRI data were obtained from the public HCP repository (https://db.humanconnectome.org) as part of the HCP 1200 data release [30]. Subjects were scanned at the Washington University in St. Louis and at the Northwestern University on Siemens 3T Tim Trios using a 12-channel head coil.

A 3D T1w magnetization-prepared rapid gradient-echo sequence (MPRAGE) and a 3D T2w sampling perfection with the application of an optimized contrast using a different angle evolutions sequence was acquired (SPACE) were acquired at 0.7 mm isotropic resolution. Both scans were acquired sagittally. Diffusion-weighted data were obtained using three different gradient tables, with each table acquired once with right-to-left and left-to-right phase encoding polarities, respectively. Each gradient table includes approximately 90 diffusion weighting directions plus 6 *b* = 0 acquisitions interspersed throughout each run. Diffusion weighting consisted of three shells of *b* = 1,000, 2,000, and 3,000 s/mm^2^ interspersed with an approximately equal number of acquisitions on each shell within each run. MRI scanning was always preceded by a mock scanner session to allow the subjects to acclimatize to the scanner environment. Full scanning details and parameters are available at https://humanconnectome.org/storage/app/media/documentation/s1200/HCP_S1200_Release_Reference_Manual.pdf.

### MRI pre-processing

Pre-processed cortical myelin maps, as well as minimally pre-processed diffusion weighted data, were downloaded from the HCP consortium database (https://db.humanconnectome.org/) [32]. Such myelin maps were generated by the HCP consortium by using the T1-weighted and T2-weighted contrasts as described in detail elsewhere [33]. This means that the T1-weighted and T2-weighted volumes used for cortical myelin map estimation had been pre-processed according to the standard, state-of-the-art HCP pipeline [32]. This pipeline uses FreeSurfer to generate white, pial, and mid-thickness surfaces, which are then mapped to the 164 k vertex fs_LR mesh using caret and the Connectome workbench. Within this pre-processing context, the T2-weighted image is registered to the T1-weighted image using FSL’s FLIRT algorithm through a rigid body transformation and using mutual information as to cost function. Notably, the ratio of the two contrasts increases the sensitivity to detect intracortical myelin and simultaneously decreases bias. Given that the contrast related to myelin content (m) is approximately proportional to the intensity in the T1-weighted image and approximately inversely proportional to the intensity (1/m) in the T2-weighted image, the generated myelin contrast can be enhanced while canceling out most of the bias field. Furthermore, given that the T1-weighted and T2-weighted images are affected by uncorrelated noise, taking their ratio also results in an increased myelin contrast relative to noise (i.e. increased contrast-to-noise ratio).

The diffusion pre-processing pipeline also included intensity normalization across runs, EPI distortion correction through the TOPUP algorithm (part of FSL), eddy current and motion correction through the EDDY tools (also part of FSL), gradient non-linearity correction, calculation of gradient bvalue/bvector deviation, registration of mean b0 to native volume T1w in FLS and transformation of diffusion data, gradient deviation, and gradient directions to 1.25 mm structural space.

### MRI diffusion modeling

Pre-processed diffusion data were used to estimate both the DTI and NODDI models in all participants. In detail, b0 and b1000 data were employed to fit the diffusion tensor model to each individual, from which FA, mean diffusivity (MD), axial diffusivity (AD), and RD maps were extracted. Additionally, all multi-shell diffusion data were used to fit the NODDI model, from which the neurite density index (NDI) and orientation dispersion index (ODI) maps were computed. All diffusion modeling was performed using nonlinear optimization within the Microstructure Diffusion Tooolbox (https://github.com/robbert-harms/MDT).

### Statistical analyses of MRI diffusion data

To improve co-registration accuracy with respect to standard white-matter analysis pipelines, we created a specific and customized template from the *n* = 974 individual FA maps. All FA images where nonlinearly co-registered to each other (symmetrical diffeomorphic mapping) and averaged iteratively (*n* = 5 iterations), and finally co-registered to the template. The template creation and registration procedures were performed using the ANTs package.

Non-linear transformations were initialized through a chain of center of mass alignment, rigid, similarity, and fully affine transformations. Non-linear transformations from the individual FA images into custom template space were then applied to MD, RD, AD as well as MDI and ODI maps. Subsequent analyses followed tract-based spatial statistics procedures including thinning to create a mean FA skeleton that represents the centers of all tracts common to the group and projection of all other (MD, RD, AD, MDI, and ODI) maps onto the skeleton. This procedure further improves robustness to potential co-registration errors.

Next, the resulting maps were fed into voxel-wise intersubject statistical analyses as described in the Statistical analysis of intracortical myelin data section. In short, we investigated the associations between subject-specific diffusion indices at each voxel and individual values in sleep scores by formulating a multivariate general linear model (GLM). The regression models also included, as covariates of no interest, age, sex, body mass index, total brain volume, personality traits, and time of day at which MRI scanning occurred. To correct for multiple comparisons over space, we used permutation-based, non-parametric inference within the *randomise* tool (part of FSL). More specifically, for each comparison, we employed *n* = 10,000 permutations and the *p*-values were calculated and corrected for multiple comparisons using the “2D” parameter settings with the threshold-free cluster enhancement (TFCE) procedure, thereby avoiding the use of an arbitrary threshold for the initial cluster formation.

### Statistical analysis of intracortical myelin data

First, intracortical myelin maps for all subjects were converted to freesurfer “fsaverage” space (a 164k vertex space which represents a standard-subject, common space surface reconstruction template) for statistical inference using the Connectome workbench (https://wiki.humanconnectome.org/download/attachments/63078513/Resampling-FreeSurfer-HCP.pdf).

Next, we studied the associations between subject-specific intracortical myelin measures at each vertex and individual values in sleep scores by formulating a multi-variate GLM. The regression models included, as covariates of no interest, age, sex, education, total intracranial volume (TIV), fluid intelligence, body mass index, personality traits, and time of day at which MRI scanning occurred. All these factors can individually affect myelination and sleep, therefore they were treated as “nuisance” of confounding variables in the GLMs [29, 34–39]. Blood pressure (BP), which in itself may also affect white matter integrity [40], was not included as a covariate of no interest as its potential effect was already partially accounted for by the inclusion of BMI, to which it was significantly correlated (*r* = 0.4, *p* < 0.0001). In other words, we designed our GLMs to avoid collinearity between their variables, which can bias GLM estimation and interpretation. For the same reason, TIV was orthogonalized to sex before inclusion in the GLMs completely removing the well-known collinearity between these two variables (*r* = 0.002, *p* = 0.9 after orthogonalization). Finally, the inclusion of the time of day when the MRI scan took place was included to account for circadian effects on oligodendrocyte physiology and myelin production [37]. Subjects’ chronotype is not available on the HCP dataset.

To control for false positives as well as multiple comparisons, cluster correction was completed using Monte Carlo simulation (with a vertex-wise cluster forming the threshold of *p* < 0.001) at a cluster-wise *p*-value (CWP) of 0.05, smoothing 10 mm. This entails: (1) synthesizing a z-map, (2) smoothing the z-map, (3) thresholding at the chosen level (i.e. *p* < 0.001), (4) finding clusters in the thresholded z-map, and (5) recording the area of the largest cluster. Steps 1–5 were repeated *n* = 10,000 times, which gives rise to an *n*-sample distribution estimate of the maximum cluster size under the null hypothesis.

Finally, for each cluster found after thresholding of the original data, a *p*-value was assigned which corresponds to the probability of seeing a cluster of that size or larger during the simulation. Whenever we detected statistically significant effects, we also calculated vertex-wise effect size maps by deriving partial correlation coefficients directly from the GLM which was fitted for each regressor/contrast.

### Structural equation modeling

**Figure 2.**
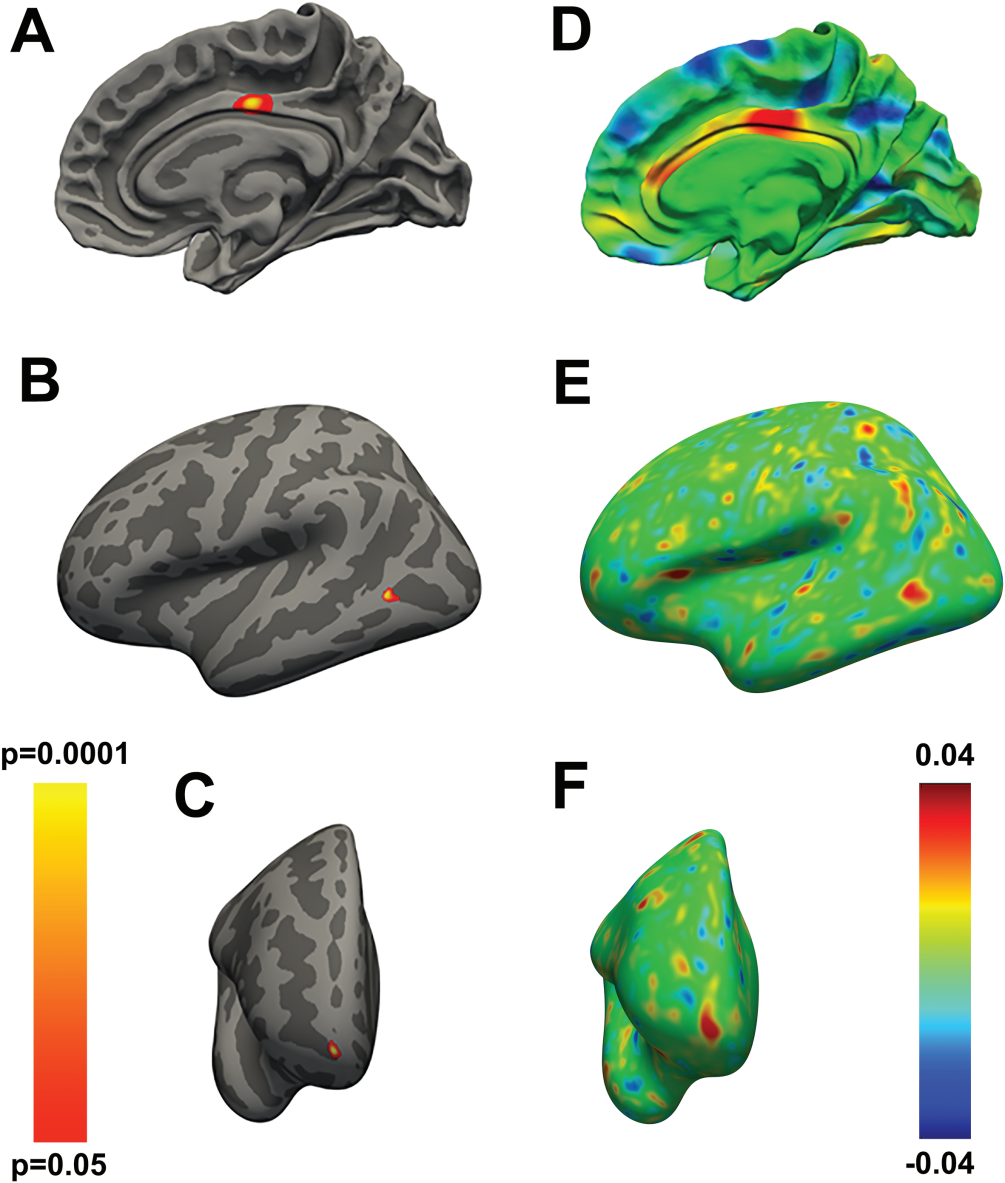
(A) Vertex-wise statistical map showing the *positive* association between the quantity of sleep and the regionally specific intracortical myelin content in the mid-posterior cingulate cortex. (B, C) Vertex-wise statistical map showing the *negative* association between the quality of sleep and the regionally specific intracortical myelin content in the posterior middle temporal gyrus (B) and anterior orbitofrontal cortex (C). (D–F) Effect size maps for the findings respectively presented in panel A–C. The color bar on the left represents the *p*-values for the maps shown in A–C, while the color bar on the right represents the strength of the effect sizes for the maps displayed in D–F. Covariates of no interest in the GLMs employed to generate the maps in A–F were sex, age, education, body mass index, fluid intelligence, TIV, personality traits as assessed via the NEO-FFI, and time of day when the MRI scanning occurred.

To characterize the direction of the association between self-reported sleep amount and quality, intracortical myelin, and self-report measures of emotional behavior, we used structural equation modeling (SEM) as implemented with the IBM© SPSS© AMOS package. Starting from the results of surface-based analyses, we built models that included subject-wise myelin data (extracted from regions in which we detected a significant association with sleep quality and quantity), sleep amount and quality and emotional variables. The model also included latent unobservable variables which separately influenced myelin content, sleep, and emotional behavior (see Figure 2).

## Results

### Sleep measures

Out of the approximately *n* = 1,200 original sample from the HCP, we employed all participants for which a T1/T2, as well as valid DTI MRI data, was available, resulting in *n* = 974. The mean age was 28.7 ± 3.7 years and *n* = 530 participants were women (Table 1). The Pittsburgh Sleep Quality Index (PSQI) questionnaire was used to assess sleep quality and duration [31]. The average PSQI total score was 4.8 ± 2.8 with *n* = 305 participants (31.3%) scoring >5 (indicating “poor sleep quality”). The average sleep duration was 6.8 ± 1.1 h (Figure 1, Table 2).

### Chronic short sleep and poor sleep quality relate to negative emotionality but not cognitive performance

Reduced sleep quality or quantity has been repeatedly associated with worse cognitive performance and increased emotional lability [2, 4, 5]. To confirm this previously reported finding in our dataset, we employed bivariate correlations with Bonferroni correction for multiple comparisons to test for the relationship between performance during neuropsychological tasks and PSQI scores [31]. We also examined the association between PSQI scores and self-reported measures of negative emotionality, positive affect, perceived levels of stress, and life satisfaction (Supplementary Table S1).

We found that both the amount and quality of sleep were weakly and generally not significantly associated with cognitive performance. In contrast, the amount and quality of sleep were both significantly associated with emotional measures (Table 3). More specifically, chronic short sleep or bad sleep quality was significantly associated with higher levels of anger, fear, perceived stress, and lower levels of life satisfaction and positive affect (Table 3). We also found a significant correlation between worse or shorter sleep and reward-based impulsivity (Table 3). Thus, emotion rather than cognition is more vulnerable to reduced sleep duration or poor sleep quality.

**Table 3.** Correlation between self-reported measures of sleep quality (PSQI_total score) and quantity (Sleep Amount) and measures of cognition (light grey) and emotional behavior (dark grey)

		PSQI_Score	Sleep Amount
	**PSQI_Score**		
	**Sleep Amount**	**−0.59**	
Cognition	Picture Sequence Memory—PicSeq	−0.02	0.03
	Cognitive Flexibility—CardSort	−0.03	0.04
	Inhibitory control—Flanker	−0.03	0.00
	Fluid Intelligence—PMAT24_A_CR	−0.10	0.11
	Fluid Intelligence—PMAT24_A_RTCR	−0.08	0.09
	Reading decoding—ReadEng	**−0.13**	**0.13**
	Vocabulary Comprehension—PicVocab	−0.12	0.10
	Processing Speed	−0.03	−0.01
	Delayed Discount—DDisc_AUC_40K	−**0.16**	**0.18**
	Spatial orientation—VSPLOT_TC	−0.04	0.07
	Spatial orientation—VSPLOT_CRTE	0.00	0.03
	Sustained Attention—SCPT_TPRT	0.07	0.01
	Sustained Attention—SCPT_SEN	−0.01	0.06
	Sustained Attention—SCPT_SPEC	−0.08	0.09
	Verbal Episodic Memory—IWRD_TOT	−0.06	0.08
	Verbal Episodic Memory—IWRD_RTC	0.03	0.04
	Working Memory—ListSort	−0.05	0.05
Emotion	Negative Affect—AngAffect	**0.29**	−**0.13**
	Negative Affect—AngHostil	0.21	−0.09
	Negative Affect—AngAggr	0.15	−0.04
	Negative Affect—FearAffect	0.31	−0.09
	Negative Affect—FearSomat	**0.26**	**−0.11**
	Negative Affect—Sadness	0.26	−0.08
	Psychological Well-being—LifeSatisf	−**0.27**	**0.16**
	Psychological Well-being—MeanPurp	−0.20	0.07
	Psychological Well-being—PosAffect	−**0.23**	**0.14**
	Social Relationships—Friendship	−0.12	0.03
	Social Relationships—Loneliness	0.21	−0.07
	Social Relationships—PercHostil	0.14	−0.05
	Social Relationships—PercReject	0.20	−0.09
	Social Relationships—EmotSupp	−0.17	0.10
	Social Relationships—Social Relationships	−0.11	0.05
	Stress and Self-Efficacy—PercStress	**0.32**	−**0.13**

Numbers indicate Pearson *r* values. Cell background is dark graded according to the strength of the correlation. Bold values indicate significant relations with both sleep measures (*p* < 0.05, Bonferroni corrected for multiple comparisons).

### Chronic short sleep and poor sleep quality do not relate to white-matter integrity

We then tested for associations between sleep amount and quality and white matter integrity. To this end, we employed whole-brain voxel-wise analyses examining the associations between DTI-derived measures, including FA, MD, AD, and RD, and the amount and quality of sleep, as measured by PSQI. These analyses did not reveal any significant correlation between sleep measures and DTI parameters.

To further explore the relationship between white matter integrity and sleep measures, we used the more recently developed NODDI model, which estimates the NDI and ODI. The NDI and ODI have been shown to offer greater sensitivity and specificity in detecting microstructural changes relative to the classic DTI measures as FA, MD, AD, and RD [15].

Nevertheless, neither NDI nor ODI indices showed significant associations with the self-reported measures of sleep quality and quantity. Together, these data suggest that self-reported amount and quality of sleep is not related to variability in white matter microstructural integrity, as indirectly assessed via a broad range of MRI measures.

### Chronic short sleep and poor sleep quality relate to lower intracortical myelin

Next, we investigated the relationship between sleep amount/quality and intracortical myelin content, as measured by the T1/T2 MRI signal ratio [26, 27]. We found a significant association (*p* = 0.038, corrected) between the amount of sleep and intracortical myelin levels in the mid-posterior cingulate cortex (Table 4, Figure 2, A and B) and between the quality of sleep and intracortical myelin content in the middle temporal cortex (*p* = 0.024, corrected) and anterior orbitofrontal cortex (OFC, *p* = 0.034, corrected) (Table 5, Figure 2, C–D). Thus, people reporting the lower amount of sleep show lower levels of intracortical myelin in the mid-posterior cingulate cortex, while people reporting poor quality of sleep manifested lower intracortical myelin in the middle temporal cortex and anterior OFC.

**Table 4.** Clusters related to positive association between the amount of sleep and intracortical myelin levels (*n* = 974, 530 females)

Region (Brodmann’s area, BA)	Amount of Sleep	Max	Size (mm^2^)	X	Y	Z	CWP
Mid-posterior Cingulate (BA23), R	Positive association	4.6	87.73	1.3	−13.1	35.2	0.038

X, Y, Z, Montreal Neurological Institute (MNI) coordinates of the local cluster-wise maxima; R, right hemisphere; Max, the maximum −log10 of the CWP.

**Table 5.** Myelin clusters showing negative association between the PSQI and intracortical myelin levels

Region (Brodmann’s area, BA)	PSQI	Max	Size (mm^2^)	X	Y	Z	CWP
Middle Temporal (BA37), L	Negative association	3.8	98.57	−58.6	−62.4	2	0.024
Frontal Pole (BA10), R	Negative association	4.2	87.04	13.1	62.9	−10.6	0.034

X, Y, Z, Montreal Neurological Institute (MNI) coordinates of the local maxima; R, right hemisphere; L, left hemisphere; Max, the maximum −log10 of the CWP.

### Pathways from sleep to emotional behavior, via intracortical myelin

**Figure 3.**
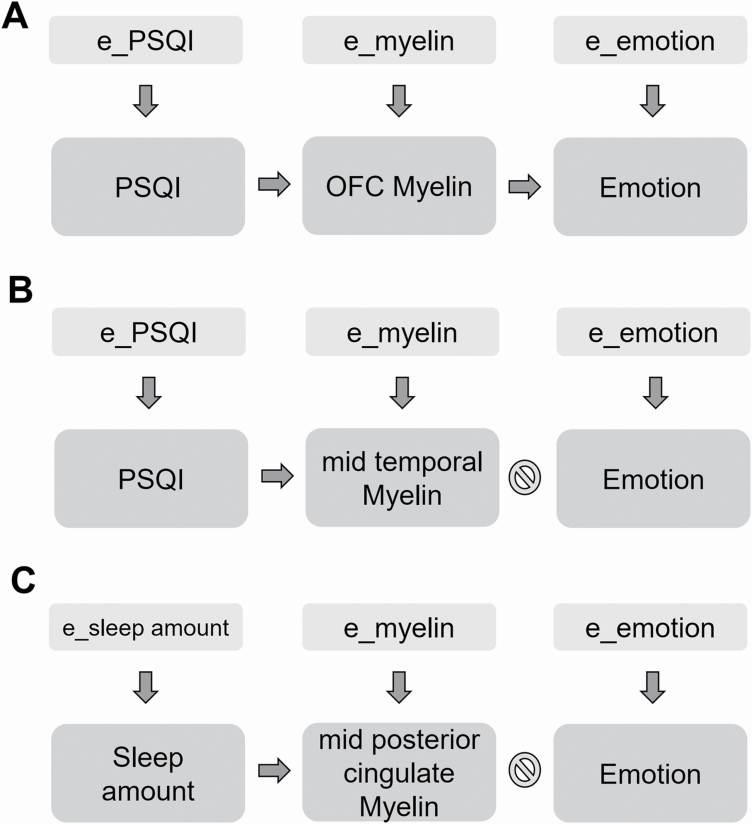
SEM showing that quality of sleep causally mediates emotional behavior by regulating the levels of intracortical myelin of anterior OFC (A), but not of middle temporal (B). (C) Same SEM showing no effect of sleep amount on emotional behavior via the regulation mid-posterior cingulate intracortical myelin. Note that each element of the SEM is also influenced by an independent latent error (*e_PSQI or e_Sleep amount*, *e_myelin*, *e_emotion*). See Table 6 for the results of the SEM analyses and associated statistics.

To further characterize the association between self-reported sleep amount and quality, intracortical myelin, and self-report measures of emotional behavior, we used SEM. The model included subject-wise myelin data extracted from the anterior OFC cluster (see Figure 2). We chose the anterior OFC because (1) this region showed a significant correlation between myelin cortical content and sleep quality, (2) it has been implicated in emotional behavior and affective control [41, 42], and (3) emotional reactivity and sleep duration and quality were significantly related in our dataset. We, therefore, tested the hypothesis that poor sleep quality enhanced anger, fear, and/or reduced positive affect via the mediating effects of intracortical myelin content in the anterior OFC (Figure 3).

**Table 6.** Relations between sleep measures (Sleep amount and quality [PSQI total score]), intracortical myelin in the regions derived from the vertex-wise analysis (mid-posterior cingulate, middle temporal, and anterior OFC), and emotional behavior variables resulting from SEM modeling

Sleep	*P* (sleep→myelin)	Myelin cluster	*P* (myelin→behavior)	Behavior
Sleep amount	**0.001 (+)**	Mid-posterior cingulate	0.503	Anger
Sleep amount	**0.001 (+)**	Mid-posterior cingulate	0.062	Fear
Sleep amount	**0.001 (+)**	Mid-posterior cingulate	0.956	Positive affect
Sleep quality	**0.001 (−)**	Middle temporal	0.495	Anger
Sleep quality	**0.001 (−)**	Middle temporal	0.2	Fear
Sleep quality	**0.001 (−)**	Middle temporal	0.696	Positive affect
Sleep quality	**0.001 (−)**	Anterior OFC	**0.01 (−)**	Anger
Sleep quality	**0.001 (−)**	Anterior OFC	0.53	Fear
Sleep quality	**0.001 (−)**	Anterior OFC	**0.007 (+)**	Positive affect

Statistically significant relations are in bold and the sign (+ or −) indicates the direction of the association (positive or negative, respectively).

We found that the effects of sleep quality on both anger (*p* = 0.01) and positive affect (*p* = 0.007) were, respectively, explained by lower and higher myelin content in the anterior OFC. In contrast, the pathway from sleep to fear (via OFC myelin content) was not significant (Table 6). To add anatomical specificity to these findings, we built additional models that included intracortical myelin data from the mid-posterior cingulum and middle temporal cortex, that is, the other two regions that were associated with sleep measures in the exploratory whole-brain analyses. These additional models did not show significant effects in the pathways linking intracortical myelin to emotional measures.

In summary, poor sleep quality increases negative emotionality and reduces positive affect via reduced levels of intracortical myelin content in the anterior OFC. Sleep dependent changes in myelin content in the mid-posterior cingulum and middle temporal cortex did not affect emotional behavior.

## Discussion

Poor quality of sleep affects the human myelocortical architecture in the limbic system which, in turn, has a negative impact on emotional behavior. More specifically, poorer quality of sleep enhanced anger feelings and decreased positive affect via the mediating effect of lower intracortical myelin in the anterior OFC, a limbic region consistently involved in anger control and emotional regulation [41]. Furthermore, scarcer quality of sleep related to lower intracortical myelin level in the anterior OFC and middle temporal cortex, while a lower amount of sleep related to lower intracortical myelin in the mid-posterior cingulate cortex.

### Emotional dysregulation but not cognitive deficit relates to poor sleep in young adults

Cognitive deficits and emotional dysregulation are typically reported by subjects experiencing sleep loss and/or reduced quality of sleep. At the cognitive level, attentional impairments and, more generally, deficits in executive functions are linked to poor sleep [1, 2]. On the other hand, poor sleep quality and quantity enhance the experience of negative emotions, reduce the occurrence of positive feelings, and alter how individuals process affective stimuli [3, 4].

Overall, our findings showed that poor quality and quantity of sleep relate to individual differences in self-reported emotions rather than to specific or general neuropsychological deficits. This selective effect may depend on the relatively young age of our participants (age-range: 22–37). This interpretation is consistent with previous data showing that some cognitive abilities as working memory are less vulnerable to sleep disruption in adolescents and young adults relative to older people [43–45]. Furthermore, the PSQI evaluated the sleeping habits only in the last month before the MRI assessment, rather than after acute sleep deprivation. This implies that participants might cope better (in cognitive terms) with chronic short sleep or poor sleep quality than after acute sleep deprivation. An alternative (but not mutually exclusive) explanation is that the degree of sleep disruption that is chronically accumulated over a month time may not be sufficient to alter the cognitive performance as such. In keeping with this speculation, other studies using similar self-report scales to the PSQI have found only modest associations between quality of sleep and cognitive deficit [46, 47].

In our sample, emotional behavior appeared particularly vulnerable to chronic sleep alterations. In this case, the relatively young age of our participants might have not played a protective role, consistently with a growing number of studies showing emotional distress in young adults who have experienced sleep loss [48–50]. For example, sleep debt in college students has been linked to depressive symptoms and a threefold risk of suicide attempts [51, 52]. Collectively, these data suggest that insufficient and poor-quality sleep may be more robustly associated with altered emotionality than cognitive dysfunction, particularly in young adults.

### Lower intracortical myelin content but not white matter integrity relates to poor sleep

First, we evaluated the association between sleep amount and quality and a broad range of MRI measures of white-matter integrity including traditional DTI indices such as FA, MD, RD, and AD or more recently introduced ODI and NDI markers from NODDI. In contrast to previous studies, we did not find any association between these indirect measures of white matter integrity and sleep duration or quality. This may depend on the different age and demographic characteristics of the populations included across studies. The mean age in our dataset was 29 years, whereas most of the previous studies reporting an effect of sleep problems on white matter integrity included populations which were >60 years old [9, 20]. It is well-known that white matter integrity declines with age and is thought to depend on age-dependent effects on the oligodendrocytes [53]. Such age-related effects of sleep deprivation may imply that, in young people, relative to older ones, white matter integrity might be more resilient to sleep loss. Alternatively, the DTI measures that we employed here may have not sufficient sensitivity to detect the white matter changes that result from poor sleep in young adults. This implies that others, more sensitive measures of white matter integrity (e.g. QA or axonal density), could reveal subtle or more nuanced alterations in the white matter integrity that are present in young people, in relation to poor sleep [22].

Second, we assessed the relationship between sleep quality and quantity and vertex-wise intracortical myelin and found that chronic short sleep and poor sleep quality related to cortical myelin levels in three brain regions (discussed in the Effects of poor sleep on regional intracortical myelin content section). Aside from methodological differences between the intracortical myelin and white matter integrity analyses, the reasons why MRI markers of intracortical myelin, relative to MRI measures of white-matter integrity might be better predictors of quality and quantity of sleep remain to be elucidated.

Nevertheless, we speculate that this discrepancy may depend on the anatomical and functional differences between intracortical myelin and its white matter equivalent. Typically, the axons within the neocortex are only intermittently myelinated and have thus long axonal segments devoid of myelin. In contrast, axons in the white-matter tracts show a denser and more diffused pattern of myelination than the neocortex [54]. The discontinued myelination pattern in the neocortex suggests additional functions for intracortical myelin, over and above the maximization of the conduction velocity—this myelo-architectonic peculiarity may be pivotal for the precise spike timing and the optimal information transmission in the cerebral cortex [55]. It is also possible that the incomplete pattern of cortical myelination offers greater flexibility in terms of “tuning” cortical activity, which however would come at the cost of a greater susceptibility to damage as compared to white-matter myelin.

In other words, small deviations in sleep quality like the ones observed in our participants (PSQI total score 4.8 ± 2.8, mean ± *SD*), may not be sufficiently severe to alter the dense and diffuse pattern of myelination in the white matter, but may be enough to disrupt the more “scattered” and incomplete myelination in the cortical mantle. Consistently with this hypothesis, one of our study in mice showed that the axons of two white matter tracts did not show any changes in myelination after 8 h of sleep deprivation, while they showed a myelin reduction only after almost 5 days of intense sleep restriction (i.e. with a total sleep loss of about 75%) [7]. This indicates that a strong regime of sleep disruption is needed to disrupt the white-matter myelination, whereas milder sleep disruption is likely to affect the “frailer” intracortical myelin. In further keeping with this interpretation, several DTI studies have reported reduced white matter integrity in primary insomnia and obstructive sleep apnea, two sleep disorders characterized by severe chronic sleep disruption, often in elderly people [56–58].

### Effects of poor sleep on regional intracortical myelin content

First, the self-reported amount of sleep positively related to intracortical myelin content in the mid-posterior cingulate cortex. This densely connected and metabolically demanding brain region has been implicated in arousal and “balancing” externally and internally directed attention [59]. The mid-posterior cingulate also plays a key role in the propagation of sleep slow-waves and in connecting anterior frontal regions (e.g. the anterior cingulate, middle/inferior frontal gyri) to posterior parietal areas as the precuneus. The mid-posterior cingulate thus forms a “highway” for sleep slow waves traveling along the anterior–posterior axis [60]. Optimal levels of intracortical myelin in this region are therefore hypothesized to enable propagation of slow waves toward posterior regions of the brain. Moreover, the mid-posterior cingulate cortex is part of the default mode network (DMN), a circuit that has been consistently identified in resting-state functional MRI studies [61, 62]. Interestingly, spontaneous activity within the DMN is impaired after sleep deprivation [63] and subjects whose mood appear more vulnerable to sleep deprivation show lesser compact axonal pathways within this network [22]. Hence, we speculate that different levels of intracortical myelin within the mid-posterior cingulate cortex may underlie the functional integrity of the DMN that has been identified in previous studies.

Second, poor sleep quality related to lower intracortical myelin in the posterior middle temporal gyrus. This region is important for language, visuospatial skills, and multi-modal sensory integration [64]. In a positron emission tomography study, the same temporal area that we have identified here showed decreased glucose metabolism after sleep deprivation, alongside other frontoparietal regions [65]. The lower grey-matter volume of the temporal cortex has also been found in people with obstructive sleep apnea [66]. Collectively, these findings indicate a possible sensitivity of the temporal cortex to poor sleep.

Third, poor sleep quality related to lower intracortical myelin in the anterior OFC. The OFC represents the affective value of reinforcers and is undoubtedly involved in emotional regulation [41]. The OFC receives input from different sensory modalities and other limbic regions such as the amygdala and hippocampus [67]. These reciprocal pathways link sensory and affective stimuli to contextualize action generation and behavioral control. During sleep, in conjunction with the amygdala and anterior cingulate cortex, the OFC orchestrates the processing of emotional events that had occurred during the daytime [68, 69].

Consistent with previous studies showing that sleep loss increases irritability, emotional volatility, and aggression [70–73], we, respectively, found a positive and negative association between the PSQI (where higher scores reflect poorer sleep quality) and anger feelings and positive affect. Of note, lower intracortical myelin in the anterior OFC mediated both the enhancing effect of poor sleep quality on anger feelings and its dampening role on positive affect. Surprisingly, the effect of poor sleep quality on heightened fear was not mediated via the myelin content in the anterior OFC. Nevertheless, as the models including other regions did not yield significant associations, we conclude that the influence of intracortical myelin on emotional behavior was rather specific to the anterior OFC.

### Strength and limitations

The strength of our study resides in the use of a large and well-characterized sample of participants in terms of behavioral, cognitive, and demographic features (*n* = 974 people, age-range: 22–37). In addition, this study used standardized imaging analyses and pooled multiple modalities (concomitant use T1-weighted and T2-weighted MRI for myelin estimation as well as diffusion-weighted imaging for examining white matter integrity) and models (estimation of both the DTI model and the NODDI model) to assess the relationships between brain myelination and sleep duration and quality.

We also acknowledge six main limitations of our study. First, its cross-sectional design does not allow to make strong causal conclusions, although the mediation analyses provided useful insights into this regard. Second, the current sample was mainly composed of young, well-educated, and healthy individuals, which may limit the generalizability of our findings to wider and more representative populations. Third, possible errors in the surface reconstruction might have affected the estimation of the myelin content in heavily myelinated regions such as the primary motor and sensory cortices, although we note that our results were outside these brain regions. Fourth, as many other studies, we employed self-report measures of sleep amount and quality which inevitably depend on people’s judgment on their own sleeping pattern. In addition, previous research has shown that self-reported questionnaires may underestimate the sleep duration [74, 75], although this bias is mitigated in studies employing large samples [76]. Fifth, we could not rule out that some of the participants, particularly those scoring high on the PSQI, had sleep apnea, a common sleep disorder that can in itself reduce grey-matter volume and myelination [77]. However, the potential biasing effects of sleep apnea on our findings were ameliorated by including the BMI as a “nuisance” variable in the GLMs. The BMI is a significant risk factor for this sleep disorder as it strongly and positively associates with it [78]. In other words, the inclusion of the BMI as a confounding factor in the statistical models was likely to have mitigated the effects of sleep apnea on our results. Sixth, although the SEM analyses indicated that poor sleep quality related to emotional behavior via the myelination levels in the anterior OFC, SEM has limited power in cross-sectional non-interventional studies. Thus, causal investigations in animals will be indispensable to corroborate the pathways across sleep alterations, myelin content, and negative emotionality.

## Conclusions

In young adults, affective behavior is more vulnerable than cognition to poor quality and quantity of sleep. These sleep alterations also related to lower intracortical myelin content but not white matter microstructural integrity. Finally, intracortical myelin in the anterior OFC, but not other regions, mediated the association between poor sleep quality and higher negative emotionality (or lower positive affect). This study confirms that good quality of sleep is crucial for brain myelination and emotional well-being.

## Supplementary Material

zsaa146_suppl_Supplementary_File
