# Supplementary material for: Sleep quality relates to emotional reactivity via intracortical myelination
Source: Sleep. 2020 Aug 8;44(1):zsaa146. doi: 10.1093/sleep/zsaa146 (PMC7819832; doi:10.1093/sleep/zsaa146)
Supplement: zsaa146_suppl_Supplementary_File [file zsaa146_suppl_supplementary_file.docx]

**Supplementary File.** List of the tests used in the study (from the updated version of the HCP Data Dictionary for the 1200 Subjects Release)

| **Test** | **Domain** | **Test abbreviation in HCP** | **Test Description** |
| --- | --- | --- | --- |
| NIH Toolbox Picture Sequence Memory Test: Age-Adjusted Scale Score | Episodic Memory (Picture Sequence Memory) | PicSeq_AgeAdj | NIH Toolbox Picture Sequence Memory Test (PSMT): Age-Adjusted Scale Score. Participant score is normed using the age appropriate band of Toolbox Norming Sample (bands of ages 18-29, or 30-35), where a score of 100 indicates performance that was at the national average and a score of 115 or 85, indicates performance 1 SD above or below the national average for participants age band. The PSMT is an assessment of episodic memory for ages 3-85 years which involves the acquisition, storage and effortful recall of new information. It is considered a strong 'fluid ability' measure, with performance reaching a peak in early adulthood and declining across the life span. It involves recalling increasingly lengthy series of illustrated objects and activities that are presented in a particular order on the computer screen. |
| NIH Toolbox Dimensional Change Card Sort Test: Age-Adjusted Scale Score | Executive Function/Cognitive Flexibility (Dimensional Change Card Sort) | CardSort_AgeAdj | NIH Toolbox Dimensional Change Card Sort (DCCS) Test: Age-Adjusted Scale Score. Participant score is normed using the age appropriate band of Toolbox Norming Sample (bands of ages 18-29, or 30-35), where a score of 100 indicates performance that was at the national average and a score of 115 or 85, indicates performance 1 SD above or below the national average for participants age band. The DCCS is a measure of executive function, specifically tapping cognitive flexibility for ages 3-85. DCCS is considered a 'fluid ability' measure, with performance generally increasing through childhood and then declining across the adult age span. Target pictures are presented that vary along two dimensions (e.g., shape and color). Participants are asked to match a series of bivalent test pictures (e.g., yellow balls and blue trucks) to the target pictures, first according to one dimension (e.g., color) and then, after a number of trials, according to the other dimension (e.g., shape). Switch trials are also employed, in which the participant must change the dimension being matched, thus requiring the cognitive flexibility to quickly choose the correct stimulus. Scoring is based on a combination of accuracy and reaction time. |
| NIH Toolbox Flanker Inhibitory Control and Attention Test: Age-Adjusted Scale Score | Executive Function/Inhibition (Flanker Task) | Flanker_AgeAdj | NIH Toolbox Flanker Inhibitory Control and Attention Test: Age-Adjusted Scale Score. Participant score is normed using the age appropriate band of Toolbox Norming Sample (bands of ages 18-29, or 30-35), where a score of 100 indicates performance that was at the national average and a score of 115 or 85, indicates performance 1 SD above or below the national average for participants age band. The Flanker is a measure of executive function, specifically tapping inhibitory control and attention for ages 3-85. It is considered a fluid ability measure (i.e. measures the capacity for new learning and information processing in novel situations) in which performance reaches a peak in early adulthood, then tends to decline across the life span. The test requires the participant to focus on a given stimulus while inhibiting attention to stimuli (arrows for ages 8-85) flanking it. Sometimes the middle stimulus is pointing in the same direction as the flankers (congruent) and sometimes in the opposite direction (incongruent). Scoring is based on a combination of accuracy and reaction time. |
| Penn Progressive Matrices: Number of Correct Responses (PMAT24_A_CR) | Fluid Intelligence (Penn Progressive Matrices) | PMAT24_A_CR | Penn Matrix Test (PMAT): Number of Correct Responses. The PMAT measures fluid intelligence via non-verbal reasoning using an abbreviated version of the Raven's Progressive Matrices Form A developed by Gur and colleagues (Bilker et al. 2012). Participants are presented with patterns made up of 2x2, 3x3 or 1x5 arrangements of squares, with one of the squares missing. The participant must pick one of five response choices that best fits the missing square on the pattern. The task has 24 items and 3 bonus items, arranged in order of increasing difficulty. However, the task discontinues if the participant makes 5 incorrect responses in a row. |
| Penn Progressive Matrices: Median Reaction Time for Correct Responses (PMAT24_A_RTCR) | Fluid Intelligence (Penn Progressive Matrices) | PMAT24_A_RTCR | Penn Matrix Test (PMAT): Median Reaction Time (RT) for Correct Responses. The RT measure can be combined with the Correct Responses measure to gauge PMAT efficiency. The PMAT measures fluid intelligence via non-verbal reasoning using an abbreviated version of the Raven's Progressive Matrices Form A developed by Gur and colleagues (Bilker et al. 2012). Participants are presented with patterns made up of 2x2, 3x3 or 1x5 arrangements of squares, with one of the squares missing. The participant must pick one of five response choices that best fits the missing square on the pattern. The task has 24 items and 3 bonus items, arranged in order of increasing difficulty. However, the task discontinues if the participant makes 5 incorrect responses in a row. |
| NIH Toolbox Oral Reading Recognition Test: Age-Adjusted Scale Score | Language/Reading Decoding (Oral Reading Recognition) | ReadEng_AgeAdj | NIH Toolbox Oral Reading Recognition Test: Age-adjusted Scale Score. Participant score is normed using the age appropriate band of Toolbox Norming Sample (bands of ages 18-29, or 30-35), where a score of 100 indicates performance that was at the national average and a score of 115 or 85, indicates performance 1 SD above or below the national average for participants age band. The Reading Test is a measure of reading decoding skill and of crystallized abilities, those abilities that are generally more dependent upon past learning experiences and consistent across the life span for ages 7-85. The participant is asked to read and pronounce letters and words as accurately as possible. The test is given via a computerized adaptive format and higher scores indicate better reading ability. |
| NIH Toolbox Picture Vocabulary Test: Age-Adjusted Scale Score | Language/Vocabulary Comprehension (Picture Vocabulary) | PicVocab_AgeAdj | NIH Toolbox Picture Vocabulary Test (PVT): Age-adjusted Scale Score. Participant score is normed using the age appropriate band of Toolbox Norming Sample (bands of ages 18-29, or 30-35), where a score of 100 indicates performance that was at the national average and a score of 115 or 85, indicates performance 1 SD above or below the national average for participants age band. The PVT is a measure of general vocabulary knowledge for ages 3-85 and is considered to be a strong measure of crystallized abilities (those abilities that are more dependent upon past learning experiences and are consistent across the life span). The participant is presented with an audio recording of a word and four photographic images on the computer screen and is asked to select the picture that most closely matches the meaning of the word. The measure is presented in computerized adaptive format (what is presented is dependent on the participants previous responses). |
| NIH Toolbox Pattern Comparison Processing Speed Test: Age-Adjusted Scale Score | Processing Speed (Pattern Completion Processing Speed) | ProcSpeed_AgeAdj | NIH Toolbox Pattern Comparison Processing Speed Test (PCT): Age-Adjusted Scale Score. Participant score is normed using the age appropriate band of Toolbox Norming Sample (bands of ages 18-29, or 30-35), where a score of 100 indicates performance that was at the national average and a score of 115 or 85, indicates performance 1 SD above or below the national average for participants age band. The PCT is a measure of speed of processing, which is considered a 'fluid ability' because it steadily improves (time to complete task decreases) throughout childhood and adolescence, then begins to decline in adulthood. This test for ages 7-85 asks participants to discern whether two side-by-side pictures are the same or not. The items are designed to be simple to most purely measure processing speed. |
| Delay Discounting: Area Under the Curve for Discounting of $40,000 (DDisc_AUC_40K) | Self-regulation/Impulsivity (Delay Discounting) | DDisc_AUC_40K | Delay Discounting: Area Under the Curve (AUC) for Discounting of $40,000. A summary measure of Delay Discounting, the AUC discounting measure provides an index of how steeply the participant discounts a delayed reward of $40,000 (Myerson et al. 2001). Delay discounting describes the undervaluing of rewards that are delayed in time. It is illustrated by the fact that humans (and other animals) will often choose a smaller immediate reward over an objectively larger, but delayed reward. |
| Variable Short Penn Line Orientation: Total Number Correct (VSPLOT_TC) | Spatial Orientation (Variable Short Penn Line Orientation Test) | VSPLOT_TC | Penn Line Orientation: Total Number Correct |
| Variable Short Penn Line Orientation: Median Reaction Time Divided by Expected Number of Clicks for Correct (VSPLOT_CRTE) | Spatial Orientation (Variable Short Penn Line Orientation Test) | VSPLOT_CRTE | Penn Line Orientation: Median Reaction Time Divided by Expected Number of Clicks for Correct Trials |
| Short Penn Continuous Performance Test: Median Response Time for True Positive Responses (SCPT_TPRT) | Sustained Attention (Short Penn Continuous Performance Test) | SCPT_TPRT | Short Penn CPT Median Response Time for True Positive Responses |
| Short Penn Continuous Performance Test: Sensitivity = SCPT_TP/(SCPT_TP + SCPT_FN) (SCPT_SEN) | Sustained Attention (Short Penn Continuous Performance Test) | SCPT_SEN | Short Penn CPT Sensitivity = SCPT_TP/(SCPT_TP + SCPT_FN) |
| Short Penn Continuous Performance Test: Specificity = SCPT_TN/(SCPT_TN + SCPT_FP) (SCPT_SPEC) | Sustained Attention (Short Penn Continuous Performance Test) | SCPT_SPEC | Short Penn CPT Specificity = SCPT_TN/(SCPT_TN + SCPT_FP) |
| Penn Word Memory Test: Total Number of Correct Responses (IWRD_TOT) | Verbal Episodic Memory (Penn Word Memory Test) | IWRD_TOT | Penn Word Memory: Total Number of Correct Responses |
| Penn Word Memory Test: Median Reaction Time for Correct Responses (IWRD_RTC) | Verbal Episodic Memory (Penn Word Memory Test) | IWRD_RTC | Penn Word Memory: Median Reaction Time for Correct Responses |
| NIH Toolbox List Sorting Working Memory Test: Age-Adjusted Scale Score | Working Memory (List Sorting) | ListSort_AgeAdj | This task assesses working memory and requires the participant to sequence different visually- and orally presented stimuli. Pictures of different foods and animals are displayed with both a sound clip and written text that name the item. The task has two different conditions: 1-List and 2-List. In the 1-List condition, participants are required to order a series of objects (either food or animals) in size order from smallest to largest. In the 2-List condition, participants are presented both food and animals and are asked to report the food in size order, followed by the animals in size order. Children ages 3-6 have four practice items in each condition: two practice items in which the images appear simultaneously on the screen and two practice items in which the images briefly 'flash' sequentially on the screen. Participants ages 7-85 have two practice items, both 'flashing' in each condition. Different instructions are provided for 3-6 and 7-85 year olds in English and for 3-6, 7-17 and 18-85 year olds in Spanish. |
| NIH Toolbox Anger-Affect Survey: Unadjusted Scale Score | Negative Affect (Sadness, Fear, Anger) | AngAffect_Unadj | Anger is a concept within the Negative Affect subdomain of Emotion. Anger is characterized by attitudes of hostility and cynicism and is often associated with experiences of frustration impeding goal-directed behaviour. For adult self-report (ages 18 and above), Anger is comprised of three components: anger as an emotion, aggression as a behavioural component, and hostility as a set of cynical attitudes and mistrust of others and their motives. The NIH Toolbox Anger-Affect Survey is a computer-adaptive test (CAT) comprised of items from the PROMIS Anger Item Bank. It assesses anger as an emotion. |
| NIH Toolbox Anger-Hostility Survey: Unadjusted Scale Score | Negative Affect (Sadness, Fear, Anger) | AngHostil_Unadj | Anger is a concept within the Negative Affect subdomain of Emotion. Anger is characterized by attitudes of hostility and cynicism and is often associated with experiences of frustration impeding goal-directed behaviour. For adult self-report (ages 18 and above), Anger is comprised of three components: anger as an emotion, aggression as a behavioural component, and hostility as a set of cynical attitudes and mistrust of others and their motives. The NIH Toolbox Anger-Affect Survey is a computer-adaptive test (CAT) comprised of items from the PROMIS Anger Item Bank. It assesses anger as an emotion. |
| NIH Toolbox Anger-Physical Aggression Survey: Unadjusted Scale Score | Negative Affect (Sadness, Fear, Anger) | AngAggr_Unadj | Anger is a concept within the Negative Affect subdomain of Emotion. Anger is characterized by attitudes of hostility and cynicism and is often associated with experiences of frustration impeding goal-directed behaviour. For adult self-report (ages 18 and above), Anger is comprised of three components: anger as an emotion, aggression as a behavioural component, and hostility as a set of cynical attitudes and mistrust of others and their motives. The NIH Toolbox Anger-Affect Survey is a computer-adaptive test (CAT) comprised of items from the PROMIS Anger Item Bank. It assesses anger as an emotion. |
| NIH Toolbox Fear-Affect Survey: Unadjusted Scale Score | Negative Affect (Sadness, Fear, Anger) | FearAffect_Unadj | Fear is a concept within the Negative Affect subdomain of Emotion. Fear is best characterized by symptoms of anxiety that reflect autonomic arousal and perceptions of threat. The NIH Toolbox Fear-Affect Survey is a CAT comprised of items from the PROMIS Anxiety Item Bank. It assesses self-reported fear and anxious misery. |
| NIH Toolbox Fear-Somatic Arousal Survey: Unadjusted Scale Score | Negative Affect (Sadness, Fear, Anger) | FearSomat_Unadj | Fear is a concept within the Negative Affect subdomain of Emotion. Fear is best characterized by symptoms of anxiety that reflect autonomic arousal and perceptions of threat. The NIH Toolbox Fear-Somatic Arousal Survey is a 6-item calibrated scale comprised of items from the Mood and Anxiety Symptom Questionnaire. It assesses somatic symptoms related to arousal. |
| NIH Toolbox Sadness Survey: Unadjusted Scale Score | Negative Affect (Sadness, Fear, Anger) | Sadness_Unadj | Sadness is a concept within the Negative Affect subdomain of Emotion. Sadness is distinguished by low levels of positive affect and comprised of symptoms that are primarily affective (poor mood) and cognitive (negative perceptions of self, the world, and the future) indicators of depression. The NIH Toolbox Sadness Survey is a CAT comprised of items from the PROMIS Depression Item Bank. |
| NIH Toolbox General Life Satisfaction Survey: Unadjusted Scale Score | Psychological Well-being (Positive Affect, Life Satisfaction, Meaning and Purpose) | LifeSatisf_Unadj | Life Satisfaction is a concept within the Psychological Well-Being subdomain of Emotion. Life Satisfaction is one's cognitive evaluation of life experiences and is concerned with whether people like their lives or not. Life satisfaction includes both general (e.g., my life is going well) and domain-specific (e.g., I am satisfied with my family life) aspects. This self-report measure for adults (ages 18 and above) is a 10-item calibrated scale comprised of items from the Satisfaction with Life Scale and the Students' Life Satisfaction Scale. It assesses global feelings and attitudes about one's life. |
| NIH Toolbox Meaning and Purpose Survey: Unadjusted Scale Score | Psychological Well-being (Positive Affect, Life Satisfaction, Meaning and Purpose) | MeanPurp_Unadj | Meaning and Purpose is a concept within the Psychological Well-Being subdomain of Emotion. Meaning and Purpose is characterized by the extent to which people feel their life matters or makes sense. This self-report measure for adults (ages 18 and above) is a CAT comprised of items from the Meaning in Life Questionnaire, the Life Engagement Test, the MHI, and the FACIT-Sp. |
| NIH Toolbox Positive Affect Survey: Unadjusted Scale Score | Psychological Well-being (Positive Affect, Life Satisfaction, Meaning and Purpose) | PosAffect_Unadj | Positive Affect is a concept within the Psychological Well-Being subdomain of Emotion. Positive Affect can be described as feelings that reflect a level of pleasurable engagement with the environment such as happiness, joy, excitement, enthusiasm, and contentment. This parent-report measure (for children ages 8-12) is a CAT comprised of items from the PANAS-X. It assesses both activated (i.e., happiness, joy) as well as unactivated (i.e., serenity, peace) aspects of positive affect. |
| NIH Toolbox Friendship Survey: Unadjusted Scale Score | Social Relationships (Social Support, Companionship, Social Distress, Positive Social Development) | Friendship_Unadj | Companionship is a concept within the Social Relationships subdomain of Emotion. Companionship is characterized by self-reported perceptions of the availability of friends or companions with whom to interact or affiliate (i.e., friendship) and that one is alone, lonely or socially isolated from others (i.e., loneliness). The NIH Toolbox Friendship Survey is a self-report measure for adults (ages 18 and above) comprised of 5 calibrated items. |
| NIH Toolbox Loneliness Survey: Unadjusted Scale Score | Social Relationships (Social Support, Companionship, Social Distress, Positive Social Development) | Loneliness_Unadj | Companionship is a concept within the Social Relationships subdomain of Emotion. Companionship is characterized by self-reported perceptions of the availability of friends or companions with whom to interact or affiliate (i.e., friendship) and that one is alone, lonely or socially isolated from others (i.e., loneliness). The NIH Toolbox Loneliness Survey is a self-report measure for adults (ages 18 and above) comprised of 8 calibrated items. |
| NIH Toolbox Perceived Hostility Survey: Unadjusted Scale Score | Social Relationships (Social Support, Companionship, Social Distress, Positive Social Development) | PercHostil_Unadj | Social Distress is a concept within the Social Relationships subdomain of Emotion. Social distress is the extent to which an individual perceives his/her daily social interactions as negative or distressing. This can include aspects of perceived hostility (e.g., how often people argue with me, yell at me, or criticize me) and perceived rejection (e.g., how often people don't listen when I ask for help, or don't pay attention to me). The NIH Toolbox Perceived Hostility Survey is a self-report measure for children and adolescents (ages 8-17) comprised of 5 calibrated items. |
| NIH Toolbox Perceived Rejection Survey: Unadjusted Scale Score | Social Relationships (Social Support, Companionship, Social Distress, Positive Social Development) | PercReject_Unadj | Social Distress is a concept within the Social Relationships subdomain of Emotion. Social distress is the extent to which an individual perceives his/her daily social interactions as negative or distressing. This can include aspects of perceived hostility (e.g., how often people argue with me, yell at me, or criticize me) and perceived rejection (e.g., how often people don't listen when I ask for help, or don't pay attention to me). The NIH Toolbox Perceived Rejection Survey is a self-report measure for children and adolescents (ages 8-17) comprised of 5 calibrated items. |
| NIH Toolbox Emotional Support Survey: Unadjusted Scale Score | Social Relationships (Social Support, Companionship, Social Distress, Positive Social Development) | EmotSupp_Unadj | Social Support is a concept within the Social Relationships subdomain of Emotion. Perceived social support is the extent to which an individual view his/her social relationships as available to provide aid in times of need or when problems arise (Cohen, 2004). This includes emotional/informational types of perceived social support for children and adolescents. Emotional Support refers to the perception that people in one's social network are available to listen to one's problems with empathy, caring and understanding, and Informational Support refers to the perception that people in one's social network are available to provide information or advice needed to solve problems that arise. This self-report measure for children and adolescents (ages 8-17) is a 7-item calibrated scale. |
| NIH Toolbox Instrumental Support Survey: Unadjusted Scale Score | Social Relationships (Social Support, Companionship, Social Distress, Positive Social Development) | InstruSupp_Unadj | Social Support is a concept within the Social Relationships subdomain of Emotion. Perceived social support is the extent to which an individual view his/her social relationships as available to provide aid in times of need or when problems arise. This includes instrumental and emotional/informational types of perceived social support. Instrumental Support refers to the perception that people in one's social network are available to provide material or functional aid in completing daily tasks (such as making meals or providing transportation) if needed. This self-report measure for adults (ages 18 and above) is an 8-item calibrated scale. |
| NIH Toolbox Perceived Stress Survey: Unadjusted Scale Score | Stress and Self Efficacy (Perceived Stress, Self-Efficacy) | PercStress_Unadj | Perceived Stress is a concept within the Stress & Self-Efficacy subdomain of Emotion. Perceived Stress is defined by individual perceptions about the nature of events and their relationship to the values and coping resources of an individual. This self-report measure for adults (ages 18 and above) is a CAT comprised of items from the Perceived Stress Scale-10. It assesses how unpredictable, uncontrollable, and overloading respondents find their lives. |
